# Meeting Report: Knowledge and Gaps in Developing Microbial Criteria for Inland Recreational Waters

**DOI:** 10.1289/ehp.0901627

**Published:** 2010-01-25

**Authors:** Samuel Dorevitch, Nicholas J. Ashbolt, Christobel M. Ferguson, Roger Fujioka, Charles D. McGee, Jeffrey A. Soller, Richard L. Whitman

**Affiliations:** 1 University of Illinois at Chicago School of Public Health, Chicago, Illinois, USA; 2 Office of Research and Development, U.S. Environmental Protection Agency, Cincinnati, Ohio, USA; 3 Ecowise Environmental, Penrith, New South Wales, Australia; 4 Australian National University, Canberra, Australian Capitol Territory, Australia; 5 University of Hawaii, Water Resources Research Center, Honolulu, Hawaii, USA; 6 Orange County Sanitation District, Fountain Valley, California, USA; 7 Soller Environmental, Berkeley, California, USA; 8 U.S. Geological Survey, Lake Michigan Ecological Research Station, Porter, Indiana, USA

**Keywords:** Clean Water Act, environmental epidemiology, environmental microbiology, indicator bacteria, inland waters, predictive modeling, waterborne pathogens, water recreation

## Abstract

The U.S. Environmental Protection Agency (EPA) has committed to issuing in 2012 new or revised criteria designed to protect the health of those who use surface waters for recreation. For this purpose, the U.S. EPA has been conducting epidemiologic studies to establish relationships between microbial measures of water quality and adverse health outcomes among swimmers. New methods for testing water quality that would provide same-day results will likely be elements of the new criteria. Although the epidemiologic studies upon which the criteria will be based were conducted at Great Lakes and marine beaches, the new water quality criteria may be extended to inland waters (IWs). Similarities and important differences between coastal waters (CWs) and IWs that should be considered when developing criteria for IWs were the focus of an expert workshop. Here, we summarize the state of knowledge and research needed to base IWs microbial criteria on sound science. Two key differences between CWs and IWs are the sources of indicator bacteria, which may modify the relationship between indicator microbes and health risk, and the relationship between indicators and pathogens, which also may vary within IWs. Monitoring using rapid molecular methods will require the standardization and simplification of analytical methods, as well as greater clarity about their interpretation. Research needs for the short term and longer term are described.

## Recreational Water Quality Criteria

### Ambient water quality criteria

Viral, bacterial, and protozoan pathogens are responsible for infectious disease outbreaks among recreators at coastal and inland surface waters (Yoder et al. 2008). To protect the public from recreational waterborne illness, the U.S. Environmental Protection Agency (EPA) established ambient water quality criteria (AWQC; U.S. EPA 1986). The epidemiologic studies on which the current AWQC are based suggest that at freshwater beaches, the rate of acute gastrointestinal illness attributable to swimming should be about 8 cases per 1,000 swimmers when monthly geometric mean (GM) density (concentration) of *Escherichia coli* is < 126 colony-forming units (CFU)/100 mL), or if the monthly GM density of enterococci is < 33 CFU/100 mL (EPA 1984). For marine waters, the gastrointestinal illness rate should be about 19 per 1,000 swimmers when monthly GM enterococci densities do not exceed 35 CFU/100 mL (U.S. EPA 1983). Additionally, single-sample maximum values were established to aid in day-to-day beach management. For freshwater beaches, these maxima are 235 CFU/100 mL for *E. coli* or 61 CFU/100 mL for enterococci; for marine beaches, the single sample maximum for enterococci is 104 CFU/100 mL. Nonetheless, final implementation guidelines for the 1986 AWQC were never issued, and prior to the passage of the Beaches Environmental Assessment and Coastal Health Act (BEACH Act 2000), only 11 states applied the criteria to their marine or Great Lakes recreational waters [herein referred to collectively as coastal waters (CWs)] (U.S. EPA 2006).

### Indicators and their limitations

Fecal indicator bacteria (FIB), such as enterococci and *E. coli*, are measured because they are thought to indicate the presence of fecal matter, and potentially pathogens, in surface waters. Compared with the pathogens that cause recreational waterborne infections, FIB are easier to detect and enumerate using well-established and inexpensive methods. Epidemiologic studies support the use of FIB as predictors of illness rates among swimmers (Wade et al. 2003).

Culture techniques for FIB require a minimum of 18–24 hr to perform. Thus, beach managers evaluate information that is, at best, 1 day old when deciding to issue swimming bans or advisories. By the time FIB results become available, the microbial water quality may have changed substantially (Boehm 2007; Hou et al. 2006; Whitman et al. 2004). Illness rates at beaches that are impacted by human sewage are related to FIB, which are measured by culture (U.S. EPA 1983, 1984) or by rapid molecular methods (Wade et al. 2006, 2008), but at beaches without point sources of human fecal pollution, this may not be true (Calderon et al. 1991; Colford et al. 2007). If the environmental sources and persistence of FIB were similar to those of pathogens, FIB should be good predictors of human illness. However, FIB may persist or even regrow in soil, plants, sand, and sediments (Byappanahalli and Fujioka 2004; Byappanahalli et al. 2006; Davies et al. 1995; Desmarais et al. 2002)—environments that, without a suitable host organism, do not support the replication of human viral or protozoan pathogens. Boehm et al. (2009) recently reviewed these and other limitations of FIB as indicators.

### Inland water recreation, risk, and regulation

Inland flowing (riverine) waters are surface waters with a net directional current and are confined by banks and stream beds. Lacustrine waters are freshwater bodies generally confined to a basin (lakes). According to the U.S. EPA ATTAINS database (2009), the United States has > 3.5 million miles of inland flowing waters and more than 41 million acres of inland waters (IWs).

The 1986 AWQC were based almost entirely on epidemiologic studies conducted at coastal beaches, with a small portion of data coming from Keystone Lake, Oklahoma (U.S. EPA 1983, 1984). None of the supporting data were collected at rivers. Additionally, European IW recreation research is limited by the use of unique settings (Fewtrell et al. 1992; Lee et al. 1997), limited groups of participants (van Asperen et al. 1998), limited water quality measures (Ferley et al. 1989), and abbreviated reporting of illness rates (Fewtrell et al. 1994). European trials of randomized exposure to IWs have been conducted as part of the Epibathe study (European Commission 2010; Wiedenmann et al. 2006). However, the exposure, three head immersions over a 10-min period, is different from actual swimming; thus, extrapolating findings to other contexts is difficult.

The BEACH Act (2000) mandated that the 1986 criteria be applied to all U.S. marine and Great Lakes CWs, but the mandate was not extended to IWs. This Act also required that the U.S. EPA conduct epidemiologic studies at beaches to develop information for issuing new or revised criteria. As a result, the National Epidemiological and Environmental Assessment of Recreational Water (NEEAR) study (Wade 2006, 2008) has been conducted at marine and Great Lakes beaches. To address the BEACH Act requirement that the U.S. EPA develop more timely indicators of water quality, the NEEAR study used quantitative polymerase chain reaction (qPCR) tests for FIB that could produce same-day results.

In 2006, the U.S. EPA was sued by the Natural Resources Defense Council (NRDC) and others for failing to meet BEACH Act research and regulatory deadlines for recreational waters. In August 2008, the U.S. EPA entered into a consent decree (NRDC/EPA 2008), which mandated that new or revised criteria be issued by 15 October 2012. The consent decree required that the U.S. EPA “[e]valuate the applicability of NEEAR Great Lakes data to inland water.” Thus, a policy imperative exists to consider establishing IW AWQC, but the epidemiologic knowledge base for criteria development is very limited. Extending AWQC derived from studies conducted at coastal sites to IWs involves major assumptions: *a*) similar densities of FIB reflect a similar health risk in inland and coastal settings, presumably because they reflect a similar risk of exposure to pathogens of similar infectivity and virulence; *b*) hydrogeochemical differences among inland lakes, rivers, and CWs would be assumed to have nondifferential impacts on the transport and fate of indicators and pathogens; and *c*) the criteria derived from the studies conducted at sewage-impacted coastal beaches would be assumed to protect against illness in inland settings, where the dominant pollutant sources may be wildlife and/or agricultural animals.

To assess these assumptions, and more broadly, the state of the science that could support the application of coastal-derived criteria to IWs, a 3-day workshop of 31 national and international experts was convened in February 2009 by the Water Environment Research Foundation (WERF) with support from the U.S. EPA. Workshop participants identified critical knowledge gaps and outlined research needs that could be met by December 2010 (the deadline for completing research that will be reviewed in the development of the 2012 criteria) or between 2010 and 2015 (for consideration in future AWQC). A detailed report of workshop proceedings is available online (Water Environment Research Foundation 2009). In this meeting report the chairs of the working groups and the editor of the WERF report have summarized workshop highlights and added updates based on subsequent discussions.

## CWs and IWs: Superficial Similarities

General principles of hydrology, microbiology, and public health should apply across all water recreation settings. Surface waters, regardless of matrix and geologic setting, are governed by the same ecologic, hydrologic, and geographic principles. Likewise, ingesting a specific quantity of a given viable pathogen in any surface water should produce similar health risks. Consistent with this notion, health risks associated with water recreation have been identified at Great Lakes (U.S. EPA 1984; Wade et al. 2006, 2008) and U.S. marine settings (Colford et al. 2007; U.S. EPA 1983) and with IWs in Europe (Ferley et al. 1989; Wiedenmann et al. 2006). If monitoring of FIB at inland recreational waters was mandated, testing that is currently conducted for other regulatory purposes could, with sufficient resources, be expanded. Thus, at first glance, it seems that recreational criteria derived from CW studies could be applied to and implemented for IW. There are, however, important differences to consider between IWs and CWs.

## Important Differences between CWs and IWs

Several critical differences exist between CWs and IWs, which can be understood primarily as a function of the scale of water body of interest. Scale here refers to the volume, surface area, related landscape, and the flow (for flowing waters). Scale can influence watershed interactions, runoff, dilution, currents, wave height, turbulence, resuspension, and source complexity.

### Differences in pathogen source

The ultimate determinants of health risks are thought to be dependent on the dose and virulence of pathogens ingested by recreators, not whether the waters are categorized as coastal or inland. IWs are generally dominated by more rural areas and agricultural land use and are thus more likely to be affected by wildlife and livestock than are coastal watersheds. Warm-blooded animals have the potential to carry a variety of human pathogenic bacteria and protozoa and may pose human health risks (Dorner et al. 2004). For example, *Leptospira* in the urine of infected wildlife or livestock can contaminate surface waters and infect humans via skin breaks or by ingestion. U.S. outbreaks of leptospirosis have occurred in the context of IW limited- and full-contact recreation (Jackson et al. 1993; Morgan et al. 2002; Yoder et al. 2008). A recent U.S. EPA review identified several recreational outbreaks tentatively linked to wildlife and livestock sources, although definitive confirmations of animal sources have been lacking (U.S. EPA 2009). With limited dilution in inland settings, bathers themselves can become sources of fecal pathogens. Sporadic mild illness (Calderon et al. 1991) and, more conclusively, numerous outbreaks of severe disease including *E. coli* 0157:H7 (Bruce et al. 2003; Keene et al. 1994; Yoder et al. 2008) have been linked to other bathers at IWs.

### The importance of sediment in IWs

Small lakes and streams are closely associated with watershed factors such as soils, runoff characteristics, shoreline processes, and meteorological events. Turbulent flow in IWs may lead to resuspension of sediment-associated FIB. Numerous studies have found that *E. coli* and enterococci can persist and potentially regrow in sediments and soils (Byappanahalli and Fujioka 2004; Byappanahalli et al. 2006; Davies et al. 1995; Desmarais et al. 2002). Regrowth of FIB is suspected to occur at the water/sediment boundary (Wheeler Alm et al. 2003; Yamahara et al. 2009). The effect of sediment resuspension on FIB in IWs could be amplified at the boundary layer, which, compared with CW settings, is larger in relation to the volume of surface water. An area of consensus among workshop participants is that soil and sediments, which are thought to contain proportionally fewer pathogens than fecal sources, should make larger contributions to indicator densities in surface water samples in IWs than in CW.

### Differences in hydrogeology that change indicator densities

The scale of each IW is determined by climatic conditions, geology, and ecology for that watershed. The sites for land-based contamination of IW are many, because waterborne pollutants enter IW from multiple sites as it flows downstream. Because the volumes of water in many IW sites are relatively small and land-based pollutants are often close to these sites, the dilution of pollutants is more limited in streams than in coastal settings (Olyphant et al. 2003). Parameters such as flow and turbulence vary substantially within the category of IWs and could account for more variability in FIB levels in IWs than in CWs.

### The decoupling of indicator and pathogen densities in IWs

Human pathogenic viruses and protozoan parasites reproduce in the cells of their hosts, whereas as noted above, sediments can provide favorable conditions for the persistence and re-growth of FIB. A concern among workshop participants was that a decoupling (meaning a significantly weaker association) of indicators and pathogens may occur in IWs. The basis for this concern is the combination of the known growth of FIB in sediments and the influence that sediment is thought to have on IW FIB. This decoupling is likely to result in different indicator- pathogen relationships at coastal and inland systems, as well as differences across IWs with varying hydrologic characteristics. As a result of this decoupling, FIB may overestimate pathogen densities and expected illness rates among IW recreators. In part because of this putative decoupling, the application of coastal-derived FIB criteria to inland settings should result in rates of sporadic illness (although not necessarily outbreaks of severe illness) that are at least as protective in IW as they are in CW.

## Challenges at Both CWs and IWs

### Rapid testing methods

Recent epidemiologic studies have used quantitative real time polymerase chain reaction (qPCR) measurements of enterococci (Colford et al. 2007; Wade et al. 2006, 2008), and this method may be endorsed in the new 2012 AWQC. Currently, qPCR and other rapid methods of measuring FIB are in various stages of development (Griffith et al. 2009; Noble and Weisberg 2005). If the necessary monitoring protocols and communications systems were in place, these methods could provide same-day water quality information in IWs.

One broad concern with the qPCR approach is method performance, meaning the precision, accuracy, sensitivity, and interlaboratory variability. A second concern is the interpretation of qPCR output to estimate health risks. In epidemiologic studies, enterococci qPCR results are being directly calibrated against rates of illness. That calibration is being performed at coastal beaches impacted by treated human sewage. Humic and fulvic acids found in sediment (CWs and IWs) can inhibit PCR analyses (Rutjes et al. 2006; Tsai and Olson 1992). Because of the importance of sediment in determining surface water quality in IWs, sediment may result in more inhibition than occurs in CW. The qPCR methods detect viable, nonviable, and cell-free DNA of FIB, whereas conventional methods detect culturable microbes only. The distribution of these components of the qPCR signal (Nocker et al. 2007) could be different in IW compared with CW, particularly at sites impacted by treated wastewater. IW pollutant sources and sediments may result in qPCR-illness rate relationships that are different from those described (Wade et al. 2006, 2008) in CW.

### Predictive modeling: opportunities and uncertainties in CWs and IWs

Modeling approaches offer alternatives to epidemiologic studies or extensive microbial monitoring. Simple regression modeling of FIB densities use real-time information such as meteorological and physical parameters, such as turbidity, to produce a timely and, in the long-run, a lower-cost alternative to microbiological monitoring (Boehm et al. 2007; Frick et al. 2008; Olyphant and Whitman 2004). Regression models are used to issue beach notification at three Great Lakes locations and on the Schuylkill River (Philly RiverCast 2009). Mechanistic models, which make use of microbial loading, dilution, decay, transport, and other parameters to predict location-specific densities of FIB (Boehm et al. 2005; Steets and Holden 2003), are probably best used for evaluating management practices in watersheds. These approaches model FIB levels; it is unknown whether they predict pathogen presence or illness rates.

The above approaches predict water quality, whereas quantitative microbial risk assessment (QMRA) is used to predict health risks in populations (Haas et al. 1999). The inputs to QMRA models include readily obtainable demographic and water quality data. Health risks are predicted using estimates of pathogen densities, water exposure, and dose response (number of units of pathogens ingested as a predictor of illness probability). Conversely, a desired water quality target can be modeled to meet a health risk target. QMRA allows evaluations of relative risks across a range of site-specific contamination scenarios. Few studies have directly compared QMRA projections with epidemiologic observations (Ashbolt et al. 1997), although the NEEAR study site in Boqueron, Puerto Rico (http://www.epa.gov/NHEERL/neear/) will prospectively collect information needed to compare modeled (QMRA) and observed risk. Predictive models of water quality and QMRA can be only as accurate as their inputs; some input data, such as dose response, remain limited. Sensitivity analyses can evaluate sources of uncertainty in model estimates to allow prioritization of additional data collection (such as spatiotemporal variability in indicators and pathogens).

### Policy and implementation challenges

To protect the public from waterborne illness at IWs, criteria will have to be established based on a targeted level of risk. In the absence of stakeholder input, such a targeted risk could not yet be considered an acceptable risk. The unadjusted rate of illness attributable to swimming at Great Lakes point source–impacted bathing beaches appears to be about 20–25 cases of gastrointestinal illness per 1,000 swimmers (Wade et al. 2008). In addition to the rate of illness, the severity of illness attributable to water recreation is also an important consideration in characterizing risk. The U.S. epidemiologic studies (set in CW) have described rates of gastrointestinal illness, generally thought to be mild and self-limited. By contrast, reported disease outbreaks in IWs have included rare but potentially life-threatening infections, likely due to limited dilution and proximity to fecal sources (including other bathers). Once elements of acceptable risk, rate, and severity have been defined, specific values of FIB can be evaluated with a goal of keeping risk below those levels.

AWQC developed to protect recreational water users are applied to other Clean Water Act (CWA) programs, such as the listing of impaired waters and discharge limits (under Section 303d of the CWA). A better understanding of wastewater treatment effects on the components of the qPCR signal (viable bacteria, nonviable bacteria, and cell-free DNA) will help define the value of qPCR monitoring for these other CWA purposes. Translation factors or a refinement of qPCR assays to identify viable cells (Nocker et al. 2007) may be needed to determine, for example, if treated wastewater effluent met discharge standards. The costs of implementing qPCR requirements of new AWQC for both CWs and IWs will be significant and could result in the allocation of local funds away from other water quality programs. Same-day measures of FIB have limited benefits beyond beach notification. Continued use of culture-based methods in those contexts is reasonable. A potential advantage of qPCR is the ability to differentiate human from nonhuman sources of FIB. Work published after the workshop evaluated numerous promising approaches for both rapidly evaluating microbe density and differentiating human from nonhuman sources (Griffith et al. 2009). However, limitations of specific qPCR approaches for differentiating sources has been described (Stapleton et al. 2009).

New monitoring requirements for recreational waters may encompass the vast number of inland lakes and miles of rivers within individual states. The application of predictive models, sanitary surveys, and QMRA to develop site-specific standards, particularly where the dominant source of fecal pollution is nonhuman, is of interest to the regulated community. It remains an open question whether these alternatives to epidemiologic studies would provide accurate and sufficiently precise projections of FIB and health risk.

## Critical Research Questions

Four groups of critical questions should be answered regarding measures or models of water quality as a means of assessing recreational waterborne illness risk in IW (Appendix 1):

1. **Microbial indicators as predictors of pathogen exposure and health risks.** To advance our ability to model health risk, we must characterize the transport, survival, fate, and re-growth (for bacteria) of indicators and pathogens in flowing and nonflowing IW. Persistence of pathogenic bacteria needs to be better understood, as *Campylobacter, Salmonella, Shigella*, and shigatoxin-producing *E. coli* have been found on algae growing in surface waters (Ishii et al. 2006). Similarly *E. coli* O157:H7 can persist in sediments (Bruce et al. 2003). Although the dynamics of FIB have been studied in some coastal contexts (Boehm et al. 2002; Whitman and Nevers 2004), FIB spatiotemporal variability and its determinants need to be characterized in hydrologically diverse IW.
2. **Fecal pollution sources as predictors of pathogen exposure and health risk.** It is important to determine if the source of fecal pollution modifies the indicator-health association. The assumption that human fecal pollution presents the greatest health risk needs further evaluation. Prior discussions, including those at the workshop, focused on rates of illness seen among swimmers at beaches, generally mild and self-limited. Future work should also consider illness severity, which may be substantial if agricultural animals, wildlife, or other bathers are sources of pathogens at waters where dilution is limited. The health risks to recreators at IW impacted by confined animal feeding operations have not been the subject of epidemiologic studies but are a potential concern. The possibility of zoonotic waterborne viral infections should be investigated, as recent evidence supports possible zoonotic origins of some human rotaviruses (Banyai et al. 2009; Matthijnssens et al. 2009).
3. **Molecular methods for water quality testing.** Sediments, which likely contain qPCR inhibitors, FIB (viable and nonviable), and cell-free FIB DNA present a challenge to IW monitoring. It is important to know how insolation, water chemistry, wastewater treatment, hydrologic parameters, season, and water matrix differentially affect these components of the qPCR signal. Optimizing the primers and probes, particularly those that differentiate human from other sources, and establishing procedures for minimizing naturally occurring PCR inhibitors are priorities.
4. **Other approaches to predicting IW recreation health risks.** Procedures for optimizing and validating predictive models, sanitary surveys, and QMRA approaches are important for both coastal and IW. The U.S. EPA Great Lakes survey tool (http://www.epa.gov/waterscience/beaches/sanitarysurvey/) should be modifiable for IW. For QMRA and mechanistic models, inputs such as indicator and pathogen concentrations, pathogen loading estimates, and health risks posed by various animal species are needed. An uncertainty in estimating the health risk associated with livestock is the high variability of protozoa excretion rates (Ferguson et al. 2009).

## An IW Research Agenda

To provide answers to the questions identified in Appendix 1, short-term research (within 2 years) and longer-term projects (2–5 years) are needed. These questions can be addressed through targeted literature reviews, computer modeling, field sampling for environmental microbes, laboratory research on analytic methods, and human health studies. Interdisciplinary studies would compile sediment, soil, hydrology, microbiology, and health data, all of which could be used to identify predictors of health risk. Other than epidemiologic and QMRA research, these studies would not directly inform the establishment of AWQC but could advance water quality modeling and our understanding of sources of risk and uncertainty. Research agenda elements are listed in Table 1. Limited explanations of several items follow.

### Short-term research

#### Molecular tests for FIB

Rapid tests that are strongly correlated with pathogen densities are needed to support the development of improved predictive models of health risk. Pretreatment with propidium monoazide (PMA) may allow the differentiation of DNA from intact viable cells, as opposed to extracellular DNA or DNA in cells without a functioning membrane (Nocker et al. 2007). This PMA-qPCR approach has been used to demonstrate a faster decay of the qPCR signal of *Bacteroidales* compared with conventional qPCR (Bae and Wuertz 2009). PMA pretreatment or other approaches for quantifying elements of the overall qPCR signal should be evaluated for their ability to improve qPCR predictions of pathogen presence.

#### Predictive modeling of health risk

Comparison of retrospective QMRA analyses with previously conducted epidemiologic studies could lead to revisions in QMRA model assumptions and inputs to bring projected levels of risk in line with risk levels observed in epidemiologic studies. This anchoring of the QMRA predictions will enhance their scientific credibility for predicting recreational waterborne illness.

### Longer-term research

#### Modeling water quality in real time

Long-term research is required to characterize the transport, fate, and persistence of microbes and their molecular targets in sediments and soils. Understanding how these variables change as a function of solar radiation, rainfall, and biotic and hydrologic factors will advance our ability to develop mechanistic models for watershed management and set pollutant discharge limits.

#### Pollutant source as a determinant of health risk in IW

New epidemiologic studies would fill the data gap that currently precludes directly comparing the FIB-health risk relationship in coastal and IW. Ideally, several inland sites should be selected, each with a different type of dominant source of fecal pollution. Description of both illness rate and illness severity will be important to more completely characterize risk. Health data should be collected in conjunction with data on indicators and pathogens in surface waters and sediments using conventional and emerging microbial detection methods. QMRA should be performed in tandem with the epidemiologic studies.

## Conclusions

We endorse the development of science-based criteria to protect the health of those who use marine, Great Lakes, and riverine and lacustrine recreational waters. We think that the distinction of IW versus CW is of less importance than more fundamental variables such as the scale of the body of water, the source of the pollutant, and the effects of sediment, which translate into differences in the densities, transport, and fate of indicators and pathogens. Differences in these variables between IW and CW may translate into weaker indicator–pathogen and indicator–health risk relationships for IW compared with CW. It remains an open question whether sediment in IW changes the relationship between enterococci qPCR measures and health risk, which has been described at coastal beaches impacted by human fecal pollution. A challenge in addressing health risks is the imprecision in defining of risk, as frequent mild illness (seen in coastal epidemiologic studies) may be of less public health concern than infrequent severe illness (described in outbreaks of disease in IW). We suspect that the application of coastal-derived criteria should result in rates of sporadic mild illness that are no higher (and possibly lower) in IW than CW. We are concerned about outbreaks of severe disease caused by fecal matter from other bathers, wildlife, and livestock. In IW with limited dilution capacity and close proximity to sources, outbreaks of severe disease may be difficult to prevent by the application of coastal-derived criteria (this was not a conclusion of the workshop and represents the authors’ views). As critical research questions are answered, a basis will be established for developing criteria that would afford a similar level of protection in IW as in CW, at least for mild sporadic illness. The implementation of microbial monitoring of IW will be a challenge to local government agencies. Should rapid FIB monitoring, QMRA, sanitary survey data, or real-time modeling prove to be predictive of health risks in IW, these approaches could be used to protect the public from recreational waterborne illness.

## Figures and Tables

**Table 1:** Research to address critical IW criteria questions.

	Type of research required			
Critical questions	Library computer simulation	Laboratory	Field	Study overview
Determinants of indicator-pathogen relationships in IW	X	X	X	L: Advance mechanistic modeling of FIB and pathogens, supported by sampling of water, sediment and soil in diverse IW. Repeated sampling to characterize the fate, transport, persistence, and re-growth

Sources of indicators, pathogens, and health risk	X			S: Meta-analysis of epidemiologic studies to evaluate fecal pollutant source as a modifier of the indicator–health risk relationship
	X		X	S: Optimize and anchor QMRA models based on prior epidemiologic study results
	X		X	S: Develop a sanitary survey tool for use in future IW epidemiologic and QMRA studies
	X	X	X	S: Field sampling of feces from agricultural animals and wildlife to determine human pathogenic potential and dynamics
		X	X	L: Epidemiologic studies conducted at diverse IW sites, each with a different dominant source of fecal pollution (agricultural animals, wildlife, urban runoff, wastewater)

Molecular methods in IW: interpretation, standardization	X			S: Develop a database of relationships between rapid molecular-based and culture-based measures of indicator microbes described in the literature
		X	X	S: Characterize the persistence of specific molecular targets (human vs. other) in a variety of environmental settings and wastewater, looking at viable and nonintact cells and cell-free DNA
		X	X	S: Optimize, simplify, and standardize qPCR methods (particularly for source-specific markers) and other rapid methods; use these in epidemiologic studies
		X	X	L: Develop rapid methods for concentrating, identifying, and quantifying pathogens in recreational waters

Modeling health risk and real-time water quality	X		X	QMRA validation studies, S (retrospective) and L (prospective)
	X		X	S: Measure real-time physicochemical, hydrologic, meteorologic parameters, with microbes

Abbreviations: S, short term (< 2 years); L, longer term (2–5 years).

## Appendix 1 Critical questions for IW criteria development

1. Microbial indicators as predictors of pathogen exposure and health risks. What are the transport, fate, persistence, and regrowth characteristics of indicators and pathogens in climatically and hydrologically diverse IW?
2. Fecal pollution sources as predictors of pathogen exposure and health risk
  - Pathogen exposure
    - What are the prevalence, concentration, and virulence of human pathogens in fecal matter from birds, livestock, and wildlife mammals?
    - What is the likelihood that the various animal sources will pollute IW (proximity, scale)?
    - How does fecal pollutant source modify the FIB-pathogen relationship?
  - Health risk: how does fecal source modify indicator–health risk relationships?
3. Molecular methods for water quality testing
  - What are optimal test methods in terms of sensitivity, specificity, accuracy, and precision? How can PCR inhibitors best be addressed? What markers perform best for source identification?
  - The interpretation of rapid FIB measures of indicators: what are the relevant molecular targets that predict pathogen density and/or health risk in IW? Do viable cells detected by qPCR better predict risk than nonviable cells or cell-free DNA? How is the ratio of these three fractions impacted by wastewater treatment and environmental variables?
  - How can molecular methods be standardized and simplified for widespread implementation?
4. Other approaches to predicting IW recreation health risks
  - QMRA validation: how do QMRA predictions compare with epidemiologic observations? How can QMRA methods and inputs be improved?
  - Mechanistic and regressions models of water quality
    - Can coastal approaches for modeling FIB be optimized for IW?
    - Can real-time models predict pathogen exposure and health risk?
  - How can sanitary surveys, as a means of characterizing indicators, pathogens, or pollutant sources in IW, be validated and standardized?
